# Nutrition Intervention with High-Protein and β-Hydroxy-β-Methylbutyrate (HMB) Is Associated with Readmission Reduction and Cost Savings Among Patients with Malnutrition Risk

**DOI:** 10.3390/nu17223511

**Published:** 2025-11-10

**Authors:** Sigal Frishman, Ronit Doyev, Maya Ben Lassan, Alina Rosenberg, Orly Weinstein, Amy R. Sharn, Kirk W. Kerr, Suela Sulo, Lihi Godny

**Affiliations:** 1 Nutrition Department, Rabin Medical Center, Clalit Health Services, Petah Tikva 4941492, Israel; 2 Clalit Health Services, Tel Aviv 6209813, Israel; 3 Global Medical Affairs and Research, Abbott Nutrition, Tel Aviv 6935018, Israel; 4 Israel National Cancer Registry, Israel Ministry of Health, Jerusalem 9101002, Israel; 5 Israel Center for Disease Control, Israel Ministry of Health, Tel Hashomer, Ramat Gan 5262100, Israel; 6 Faculty of Medicine, Ariel University, Ariel 4077625, Israel; 7 Global Medical Affairs and Research, Abbott Nutrition, Columbus, OH 43219, USA; 8 Global Medical Affairs and Research, Abbott Nutrition, Chicago, IL 60045, USA; 9 Division of Gastroenterology, Rabin Medical Center, Petah Tikva 4941492, Israel

**Keywords:** beta-hydroxy-beta-methylbutyrate (HMB), cost-savings, hospital readmissions, malnutrition, nutritional intervention, oral nutritional supplements (ONS)

## Abstract

**Background/Objectives:** Malnutrition in hospitalized older adults is associated with increased healthcare utilization, prolonged hospitalizations, and higher readmission rates. Specialized oral nutritional supplements enriched with β-hydroxy-β-methylbutyrate (HMB-ONS) have shown benefits in preserving muscle mass, improving functional outcomes, and reducing readmission rates, yet real-world data on their effectiveness remain limited. This study evaluated the association between HMB-ONS use and hospital readmission rates, as well as healthcare costs, in patients with malnutrition or at risk of malnutrition. **Methods:** This retrospective study analyzed electronic medical records (2015–2021) of hospitalized patients at risk of malnutrition at two tertiary care hospitals in Israel. Patients receiving HMB-ONS during hospitalization were compared with those receiving standard ONS (S-ONS). Propensity score matching (PSM) was employed to reduce potential confounding due to differences in observable characteristics. Primary outcomes were readmission rates at 1-, 3-, and 6-month post-discharge. A cost analysis estimated per-patient hospitalization costs and financial savings from reduced readmissions. **Results:** Of 391,838 hospitalizations, 16,751 patients met the inclusion criteria. Patients with malnutrition or at risk of malnutrition who received HMB-ONS during hospitalization were PSM-matched to those who received S-ONS (n = 1440, 53.5% female, average age of 78.25 (±15.62) years). Patients who received HMB-ONS had significantly lower odds of readmission compared with those receiving S-ONS at 1 month (OR = 0.698; 95% CI: 0.548–0.888; *p* = 0.0034), 3 months (OR = 0.772; 95% CI: 0.623–0.958; *p* = 0.0187), and 6 months (OR = 0.780; 95% CI: 0.633–0.961; *p* = 0.0195). Based on these differences, the economic analysis estimated net cost savings of EUR 387.61 (USD 403.29) per patient for HMB-ONS versus S-ONS. **Conclusions:** HMB-ONS was associated with significantly lower readmission rates and healthcare costs compared to S-ONS in patients with malnutrition or at risk of malnutrition. These findings may support the use of specialized nutritional interventions to improve clinical outcomes and optimize hospital resource utilization in patients with malnutrition or at risk of malnutrition.

## 1. Introduction

Malnutrition is a critical global health concern, especially among older adults facing age-related physiological, social, and health challenges. Factors contributing to malnutrition include reduced appetite, dietary inadequacy, and social isolation, leading to poor intake of energy and key nutrients such as protein, calcium, zinc, and folate [1]. These challenges, compounded by social determinants of health, result in increased healthcare utilization, including primary care visits, hospitalizations, and emergency consultations, further increasing the burden of malnutrition on the healthcare system [2]. Malnutrition is a leading cause of functional decline, delayed recovery, and increased mortality among older adults, particularly those with sarcopenia and frailty. A meta-analysis found that malnutrition increased the risk of rehospitalization by 33%, mortality nearly threefold, and functional decline by 45%, while prolonging hospital stays by an average of two days [3].

Across diverse healthcare settings globally, timely nutritional interventions, including the use of oral nutrition supplements (ONSs), have been shown to mitigate these impacts by improving clinical outcomes and reducing healthcare resource use. Specialized ONS formulations enriched with β-hydroxy-β-methylbutyrate (HMB), a leucine metabolite that promotes protein synthesis and limits muscle breakdown [4,5], have demonstrated efficacy in preserving muscle mass, enhancing physical function, and improving quality of life among older adults with malnutrition or at risk of malnutrition [6,7].

Randomized controlled trials have reported that HMB-ONS improves muscle strength, handgrip strength, and functional outcomes, as shown in the SHIELD study among community-dwelling older adults at risk of malnutrition [8] and in the NOURISH trial among hospitalized older adults with malnutrition and COPD [9,10].

Observational studies from Europe have similarly highlighted the benefits of ONS and HMB-enriched supplementation, demonstrating improvements in recovery and functional outcomes in older adults following acute hospitalization and surgery [11,12]. Nonetheless, ONS remains underutilized globally [13,14], representing a missed opportunity to improve outcomes among high-risk patient populations.

Meta-analyses further support the role of HMB in improving muscle strength and physical performance among older adults and sarcopenic populations, particularly when combined with exercise [15,16]. However, there is limited real-world evidence on the effectiveness of HMB-ONS in hospitalized older adults globally and within Israel, where malnutrition-related healthcare challenges remain underexplored.

This study aimed to address this gap by evaluating the association between the use of HMB-ONS, hospital readmission rates, and healthcare costs among hospitalized patients with malnutrition or at risk of malnutrition in Israel. Using six years of hospitalization data from two large tertiary care hospitals, this analysis examined the potential of specialized nutritional interventions to reduce the burden of malnutrition and improve resource utilization in high-risk patient populations.

## 2. Methods

This study was a retrospective analysis of electronic medical records from 1 January 2015 to 31 December 2021 at two large tertiary hospitals in Israel affiliated with the Clalit Health Maintenance Organization (HMO): Rabin Medical Center–Beilinson Hospital, and Hasharon Hospital. Both hospitals are located within the same metropolitan area and adhere to uniform clinical nutrition policies, protocols, and ONS formulations. Institutional prescribing patterns are therefore highly aligned. The study population included hospitalized adult patients aged 18 and above with malnutrition or at risk of malnutrition, as identified based on clinical criteria, screening tools, and nutritional interventions.

Patients were included if they met at least one of the following criteria: received a recommendation for ONS during hospitalization; had a body mass index (BMI) of ≤20 kg/m^2^ in accordance with the institution’s screening protocol and the European Society for Clinical Nutrition and Metabolism (ESPEN) guidelines [17]; had a diagnosis of malnutrition based on International Classification of Diseases, Ninth Revision (ICD-9) codes; or had a Malnutrition Universal Screening Tool (MUST) score indicating malnutrition or risk of malnutrition.

To focus on patient populations that could tolerate ONS, exclusion criteria included patients receiving total parenteral nutrition (TPN), peripheral parenteral nutrition (PPN), or enteral nutrition (EN), and those admitted to intensive care units. Additionally, patients referred from or discharged to rehabilitation centers or nursing homes, pregnant women, and those who died during hospitalization were excluded. Of note, patients with end-stage metastatic cancer or a life expectancy of less than one year were not systematically excluded due to data limitations. Patients who were transferred within Rabin Medical Center were also excluded. Physical therapy was not part of the in-hospital nutritional intervention analyzed in this study. All included patients were hospitalized in acute-care wards, where any physiotherapy provided followed standard ward-based clinical practice rather than a predefined rehabilitation protocol. Since this analysis focused on general-purpose ONS formulations designed for malnourished polymorbid patients, patients on disease-specific ONS (e.g., diabetes- or renal-specific formulas) were excluded from the analysis to ensure a more uniform comparison.

Data extracted from the medical records included demographic, clinical, and nutritional data. Insurance coverage was defined in 3 categories: (1) Basic—provided by public health funds that included a standardized basket of services; (2) Standard—provided by the same public health funds as basic for an additional monthly fee that included an expanded choice of specialists and quicker access to surgeries; and (3) Extended—optional insurance purchased and offered full dental and optical care and wider elective surgery options. The primary objective was to compare hospital readmission rates at 1-, 3-, and 6-month post-discharge between patients receiving specialized ONS containing HMB (HMB-ONS) and those receiving standard ONS (S-ONS) during hospitalization; both groups received nutrition consultations from Registered Dietitians. Descriptive statistics were used for continuous data and categorical demographic data. Between-group comparisons were performed using the chi-squared test for categorical variables, the *t*-test for continuous variables, and the Wilcoxon test for ordinal variables. Multivariate regression models—both linear and nonlinear—were employed based on the nature and distribution of the available data. To mitigate potential confounding due to baseline differences between groups, a propensity score matching (PSM) approach was utilized (Table S1). Body mass index (BMI) was excluded from the matching process due to reliance on self-reported data. All statistical analyses were performed using SAS software (Version 9.4), with a significance threshold set at *p* ≤ 0.05. The study protocol received ethical approval from the Rabin Medical Center Institutional Review Board (313-22-RMC), approved on 30 August 2022. 

## 3. Results

From 2015 to 2021, there were a total of 391,838 hospitalizations of patients affiliated with Clalit HMO. Of those, 48,000 (12.2%) hospitalizations met the primary inclusion criteria for malnutrition. After excluding patients based on the predefined criteria, this study included a total of 16,751 patients.

### 3.1. Baseline Characteristics

The included patients were 50% female, with an average age of 72.0 ± 17.2 years and a median BMI of 24.5 kg/m^2^ (IQR: 21.5–28). (Table 1). The socioeconomic status (SES) of the patients varied, with the highest percentages in the middle SES brackets [18]: SES 6 (3077/18.37%) and SES 7 (3072/18.34%). Insurance coverage among patients was categorized as basic (3160/18.86%), standard (7943/47.42%), and extended (5648/33.72%).

Most patients were admitted to internal medicine (52.8%), followed by surgery (30.4%), geriatrics (9.6%), and oncology (7.2%). Regarding primary diagnoses, the most common conditions were oncological (12.7%), respiratory (11.1%), gastrointestinal and liver diseases (9.6%), musculoskeletal disorders (7.2%), and cardiovascular diseases (6.4%). Nearly half of the patients (49.6%) were classified under the “other” category.

### 3.2. Comparison of Baseline and Hospitalization Characteristics Across ONS Groups

Baseline and hospitalization characteristics differ across ONS groups, especially between the ONS and non-ONS groups. Days of hospitalization were significantly higher in the ONS group compared with the non-ONS group (12.16 (±11.68) vs. 3.77 (±4.04) days, *p* < 0.0001), though they were comparable between the HMB-ONS and S-ONS groups (11.06 (±9.60) vs. 11.09 (±10.49) days, *p* = 0.947). The mean age was 78.25 years in the HMB-ONS group compared with 74.75 years in the S-ONS group and 72.76 years in the combined ONS group. The mean BMI was significantly lower in the HMB group (24.15 kg/m^2^) compared with the non-ONS group (26.52 kg/m^2^, *p* < 0.0001). A higher proportion of patients in the HMB-ONS group were admitted to the geriatric department (33.5%) compared to 10.2% in the S-ONS group (*p* < 0.0001). Standard and extended insurance coverages were slightly but significantly higher in the HMB-ONS group, with 83.7% of HMB-ONS patients, compared to 80.2% in the S-ONS group (*p* = 0.0217) (Table 1).

The average intake of HMB-ONS was 290.42 ± 124.21 mL/day, corresponding to an approximate Ca-HMB dose of 2 g/day. The median time from admission to first ONS provision was 3 days, reflecting early initiation of nutritional support during hospitalization. Both study products were isocaloric (330 kcal per 220 mL); however, protein content differed (HMB-ONS 20 g/220 mL ≈ 9 g/100 mL vs. S-ONS 14 g/220 mL ≈ 6.4 g/100 mL), thus volume aligned with energy intake but not with protein intake.

### 3.3. Readmission Rates for Patients with Malnutrition

**Figure 1:**
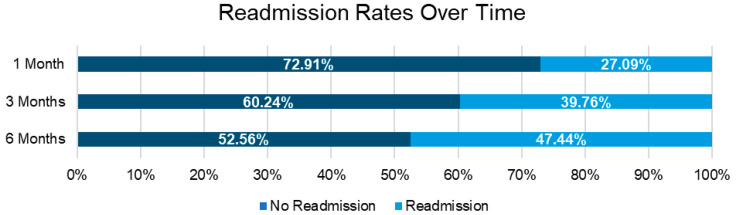
Readmission rates over time among all patients with malnutrition.

Within six months post-discharge, among all patients with and without ONS use, 52.6% had no hospital readmissions, while 47.4% had at least one readmission. Readmission rates increased over time, with 27.09% readmitted at 1 month, 39.76% at 3 months, and 47.4% at 6 months post-discharge, highlighting the progressive risk of hospital readmission among patients with or without nutritional deficits (Figure 1). As baseline and hospitalization characteristics differed substantially between ONS users and non-users, likely reflecting greater baseline clinical complexity and illness severity among those requiring ONS, we focused our analysis on a more comparable subset of patients who all received ONS, comparing the HMB-ONS and S-ONS groups. At 1 month, readmission rates were significantly lower in the HMB-ONS group (21.5%) compared with the S-ONS group (27.3%, *p* = 0.0006). Similarly, at 3 months, 33.7% of patients in the HMB-ONS group were readmitted compared with 40.4% in the S-ONS group (*p* = 0.0004). By 6 months, readmission rates in the HMB-ONS group remained lower than in the S-ONS group (41.0% vs. 48.1%, *p* = 0.0002) (Table 2).

### 3.4. Comparative Effectiveness

**Figure 2:**
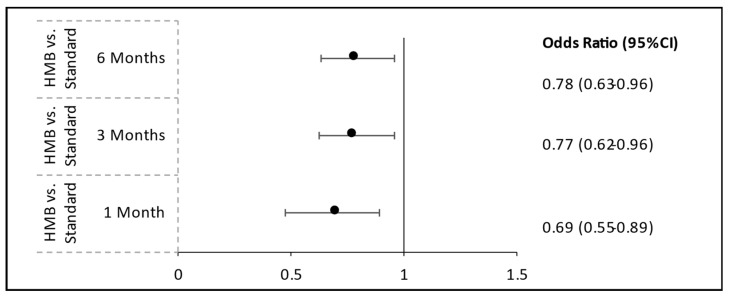
Odds ratios for hospital readmission at 1, 3, and 6 months: HMB-ONS vs. S-ONS, N = 1440. HMB-ONS, β-hydroxy-β-methylbutyrate oral nutritional supplement; ONS, oral nutritional supplement; S-ONS, standard oral nutritional supplement.

To reduce potential confounding from baseline differences between groups, we implemented PSM using key variables, including age, hospital department, volume of oral nutritional supplements administered, and length of hospital stay. A significant reduction in hospital readmission rates was observed among patients receiving HMB-ONS compared to those receiving S-ONS. At 1-month post-discharge, patients in the HMB-ONS group had significantly lower odds of readmission than those in the S-ONS group (odds ratio [OR] = 0.698; 95% confidence interval [CI]: 0.548–0.888; *p* = 0.0034). This finding persisted at 3 months, where the odds of readmission were 22.8% lower in the HMB-ONS group (OR = 0.772; 95% CI: 0.623–0.958; *p* = 0.0187). By 6 months, readmission rates remained significantly lower in the HMB-ONS group compared to the S-ONS group (OR = 0.780; 95% CI: 0.633–0.961; *p* = 0.0195) (Figure 2, Table S1).

### 3.5. Cost Analysis

A cost analysis estimated the financial implications of using S-ONS or HMB-ONS during hospitalization. For this analysis, the S-ONS cost was EUR 2.33 (Euro) (USD 2.43 (United States Dollar)) (Ensure^®^ Plus, Abbott Laboratories), and the HMB-ONS cost was EUR 2.52 (USD 2.62) (Ensure^®^ Plus Advance, Abbott Laboratories). Assuming an average length of stay of 11 days (Table 3), with patients consuming two bottles of ONS per day and receiving two dietitian consultation sessions, the cost of providing S-ONS and dietitian consultation to patients was EUR 327.6 (USD 340.86), while the cost of providing HMB-ONS and dietitian counseling was EUR 331.78 (USD 345.04). The difference between these amounts yielded an incremental cost of EUR 4.18 (USD 4.18) for HMB-ONS. Expected savings per patient from reduced readmissions were calculated by subtracting the expected cost of readmission using HMB-ONS from the expected cost of readmission using S-ONS. The average cost of hospitalization per day in Israel, based on Ministry of Health (MOH) tariffs, is EUR 804.88 (USD 837.08), and the average length of stay is 4.6 days. Using S-ONS, patients had a 6-month readmission rate of 48.1% (Table 2), which resulted in an expected cost of readmission of EUR 1780.88 (USD1852.12). To calculate the expected cost of readmission using HMB-ONS, the S-ONS readmission rate was adjusted by the reduction in likelihood of readmission in the HMB-ONS group (0.78, Figure 2), yielding an expected cost of readmission of EUR 1389.08 (USD 1444.66). Deducting the incremental cost of HMB-ONS from the expected savings due to reduced readmissions yielded net cost savings of EUR 387.61 per patient (USD 403.29).

## 4. Discussion

This is the first real-world study in Israel evaluating the potential clinical and economic impact of HMB-ONS in a large cohort of hospitalized patients with malnutrition or at risk of malnutrition. Using data from two tertiary care hospitals, this study highlighted that HMB-ONS supplementation was associated with reduced hospital readmission rates at 1-, 3-, and 6-month post-discharge by 30.2%, 22.8%, and 22.0%, respectively. Given that the expected cost of readmission is EUR 1780.88 (USD 1852.12), these reductions yielded an estimated per-patient cost savings of EUR 387.31 (USD 403.29).

### 4.1. Clinical and Functional Benefits of HMB-ONS

Malnutrition affects 3.4% of community-dwelling older adults in Israel, with ONS users representing a high-risk subgroup characterized by older age, chronic conditions, and greater healthcare utilization [2]. This study’s findings align with prior research showing that the timely provision of ONS reduced 30-day readmissions by 38.8% among hospitalized adults with malnutrition in the United States, with oncology patients experiencing an even greater reduction of 46.1%, and earlier initiation shortening hospital length of stay by 10.2% [13]. Similarly, a six-month nutritional therapy intervention in nutritionally at-risk older adults discharged from a geriatric unit in Iceland significantly reduced readmissions and shortened length of stay at all time points, with the largest difference observed at six months [19].

While interpreting these associations, it is important to note that the study products were isocaloric (330 kcal/220 mL) but differed in protein content (HMB-ONS 20 g/220 mL vs. S-ONS 14 g/220 mL); therefore, volume-based comparisons align total energy intake but do not equate protein intake, and the observed associations may reflect both protein density and the HMB component.

Beyond hospital readmissions, HMB-ONS demonstrates substantial clinical benefits. In a Singapore-based study of community-dwelling older adults at risk of malnutrition, daily HMB-ONS intake improved survival without hospital readmission, muscle strength, calf circumference, and activities of daily living [7]. Among hospitalized older adults with malnutrition and COPD in the United States, HMB-ONS use was associated with a 71% reduction in 90-day mortality, alongside significant improvements in handgrip strength and body weight [9]. Additional studies in European and Latin American populations have confirmed gains in body weight, serum albumin levels, and handgrip strength [11], as well as lean body mass, better physical function, and reduced mortality and hospital length of stay [12]. HMB supplementation has also been linked to significant gains in handgrip strength (+4.7–6.2 kg) and fat-free mass, which are critical for recovery in patients with malnutrition [20]. HMB is also known to preserve muscle mass and improve physical function in patients with cancer undergoing chemotherapy [21,22], with a meta-analysis demonstrating improvements in muscle strength and muscle mass across conditions like sarcopenia and cancer cachexia [23].

### 4.2. Mechanistic Insights

Mechanistic studies show that HMB-ONS preserves muscle fiber cross-sectional area by activating the mTOR pathway and inhibiting proteolysis via the autophagy–lysosomal and ubiquitin–proteasome systems [4,5]. However, functional improvements have been modest in patients with cirrhosis, suggesting that disease-specific metabolic disruptions may limit HMB’s efficacy [24]. HMB has also been linked to increased IGF-1 levels and reductions in inflammatory markers such as ferritin and osteopontin [22].

### 4.3. Economic Implications

HMB-ONS offers a scalable strategy to reduce the substantial healthcare burden of malnutrition, which in Israel alone accounts for 60,401 annual hospital days and USD 145.6 million in costs [25]. ONS users in Israel had significantly higher healthcare utilization, including primary care visits, dietitian consultations, and hospitalizations, reflecting the disproportionate burden among older adults with malnutrition [2]. Economic models estimate that effective ONS use could reduce overall healthcare costs by 18.9%, primarily through fewer readmissions and hospital stays [2].

In this study, HMB-ONS supplementation reduced per-patient costs by EUR 387.61 (USD 403.29 USD) compared with S-ONS, with slightly higher intervention cost offset by greater savings from reduced readmissions. International analyses further reinforce these findings. Malnutrition increases healthcare costs by 11–13% [26], while early ONS interventions reduce hospital length of stay by 10.2% in patients with malnutrition and 16.6% in ICU patients [13]. The NOURISH study reported HMB-ONS to be cost-effective, with an incremental cost of USD 398 per life-year gained and USD 524 when all costs were included. Recent 2024 models project USD 2.1 billion in annual savings post-discharge, with per-patient savings exceeding USD 1113 [27]. Similarly, a nutrition-focused Quality Improvement Program in Colombia using mainly HMB-ONS achieved a 40% reduction in healthcare utilization, 82% fewer hospitalizations, and a 64% reduction in emergency visits, with a return on investment (ROI) of USD 1.82 per dollar spent in 2022 [28].

### 4.4. Strengths and Limitations

This study provided real-world evidence from a large cohort of hospitalized patients with malnutrition or at risk of malnutrition, addressing a critical gap in nutritional research. By leveraging data from two large tertiary care hospitals in Israel, it offers insights into the potential clinical and economic impact of HMB-ONS in routine inpatient care. The use of PSM strengthened internal validity by controlling for key confounders, while an Israel-specific cost analysis enhanced the relevance of these results for local healthcare decision-making. Unlike previous trials conducted in controlled settings, this study evaluated HMB-ONS within standard hospital practice, providing pragmatic evidence that can inform clinical guidelines and policies.

Despite these strengths, several limitations should be acknowledged. The retrospective design limits causal inference, although the use of PSM mitigates some confounding. Both HMB-ONS and S-ONS were designed for polymorbid, malnourished patients and were prescribed as part of individualized dietary plans based on standardized dietitian assessments tailored to clinical condition, nutritional needs, and preferences. However, oral dietary intake beyond ONS was not systematically recorded. The ONS volume-based adjustment aligned total energy intake but not protein intake. Consequently, residual confounding by unmeasured total protein or energy intake cannot be excluded, and the observed associations may reflect a synergistic effect of higher protein density together with the HMB component rather than the isolated effect of HMB itself. Diagnostic granularity and disease-severity data were not available across all patients; thus, the admitting department was used as a proxy for clinical context in PSM. Although this approach enhanced comparability, residual confounding from unmeasured clinical factors or disease severity may persist. The relatively short in-hospital exposure to ONS limits the ability to directly attribute long-term outcomes to the intervention. Patients are usually advised to continue ONS post-discharge; however, adherence was not systematically monitored, limiting the assessment of sustained effects on 6-month readmission rates. While prior studies suggest combining HMB-ONS with resistance exercise improves functional outcomes, its feasibility in hospitalized or frail patients remains a challenge [29]. Finally, cost analyses were based on estimated hospitalization and readmission costs, warranting further research incorporating real-world post-discharge expenditures. Taken together, further randomized controlled trials and prospective cohort studies comparing the efficacy of these therapies in stratified patient populations are needed to establish causality and expand upon these findings.

## 5. Conclusions

In conclusion, these findings highlight the potential clinical and economic benefits of HMB-ONS supplementation in hospitalized patients with malnutrition or at risk of malnutrition. This study indicates that HMB-ONS may serve as an effective nutritional intervention for high-risk populations. The cost analysis further suggests that HMB-ONS is associated with reduced readmission rates and optimized hospital resource utilization, leading to cost savings of EUR 387.61 (USD 403.29) per patient. These findings highlight the contribution of quality improvement programs in improving patient outcomes and reducing healthcare costs, while providing real-world insights into the integration of HMB-ONS into routine hospital practice. Further prospective cohort studies and randomized controlled trials are warranted to validate these observations, establish causality, and identify patient subgroups most likely to benefit from HMB-ONS therapy.

## Figures and Tables

**Table 1:** Baseline and hospitalization characteristics of patients with malnutrition aby ONS group.

**Variable**	**Combined Total** ONS + Non-ONS *	**ONS**	**Non-ONS**	***p*-Value**	**HMB-ONS**	**S-ONS**	***p*-Value**
Age (years)							
N	16,751	15,650	1050	<0.0001	720	10,826	<0.0001
Mean (SD)	72 (17.2)	72.76 (16.8)	60.72 (18.85)		78.25 (15.62)	74.75 (15.79)	
Gender (%)							
Male	50	49.7	54.8	0.0012	46.5	48.7	0.2569
Female	50	50.3	45.2		53.5	51.3	
BMI (%)							
<20 kg/m^2^	10.8	10.98	8.3	<0.0001	12.97	10.98	0.1802
≥20 kg/m^2^	60.9	59.2	86.5		57.9	57.8	
Insurance Coverage (%)							
Standard + Extended	81.1	82.1	67.4	<0.0001	83.7	80.2	0.0217
Basic	18.9	17.9	32.6		16.3	19.8	
Days of Hospitalization							
N	16,751	15,699	1052	<0.0001	725	10,866	0.9466
Mean (SD)	11.6 (11.5)	12.16 (11.68)	3.77 (4.04)		11.06 (9.60)	11.09 (10.49)	
Volume Intake per Day (mL)							
N	15,368	15,368	-	-	725	10,866	0.0009
Mean (SD)	319.8 (211.8)	319.78 (211.80)	-		290.42 (124.21)	306.71 (162.21)	
**Variable**	**Combined Total** **ONS + Non-ONS**	**ONS**	**Non-ONS**	**Combined Total** **HMB + Standard**	**HMB**	**Standard**	***p*-Value**
Department (%)							
Geriatrics	9.6	9.7	3.3	11.7	33.5	10.2	<0.0001
Internal	52.8	53.0	50.0	56.3	40.3	57.4	
Surgery	30.4	29.5	44.5	25.1	23.9	25.2	
Oncology	7.2	7.6	2.2	6.9	2.3	7.2	

BMI, body mass index; HMB, β-hydroxy-β-methylbutyrate; mL, milliliters; ONS, oral nutritional supplement; SD, standard deviation; S-ONS, standard ONS. * The combined ONS group included patients who received specialized ONS formulations (e.g., elemental or disease-specific supplements) and may have had more complex clinical conditions.

**Table 2:** Readmission rates for patients with malnutrition (%) by oral nutritional supplement group.

	HMB-ONS	S-ONS	*p*-Value
1 Month ^a^	21.5	27.3	0.0006
3 Months ^a^	33.7	40.4	0.0004
6 Months ^a^	41.0	48.1	0.0002

^a^ Chi-square test, significance *p* < 0.05. HMB, β-hydroxy-β-methylbutyrate; HMB-ONS, β-hydroxy-β-methylbutyrate oral nutritional supplement; ONS, oral nutritional supplements; S-ONS, standard oral nutritional supplement.

**Table 3:** Cost analysis of S-ONS vs. HMB-ONS interventions in Euro (€) and United States Dollars (USD).

Cost Components	S-ONS		HMB-ONS	
	Euro	USD	Euro	USD
ONS Cost per Bottle	2.33	2.43	2.52	2.62
Dietitian Consultation Cost (per consultation)	138.17	143.70	138.17	143.70
Days of Hospitalization days	11		11	
Intervention Cost (ONS + Counseling)	327.6	340.86	331.78	345.04
Expected Readmission Cost	1780.88	1852.12	1389.08	1444.66
Total Cost per Patient	2108.48	2192.98	1720.86	1789.70
Net Cost Savings	–		387.61	403.29

HMB, β-hydroxy-β-methylbutyrate; ONS, oral nutritional supplements; S-ONS, standard oral nutritional supplements; USD, United States Dollar.

## Data Availability

The datasets presented in this article are not readily available due to privacy and legal reasons. Requests to access the datasets should be directed to the corresponding author.

## Supplementary Materials

The following supporting information can be downloaded at: https://www.mdpi.com/article/10.3390/nu17223511/s1, Table S1: Propensity score matching analysis.

## Abbreviations

BMI, Body Mass Index; CI, Confidence Interval; COPD, Chronic Obstructive Pulmonary Disease; ESPEN, European Society for Clinical Nutrition and Metabolism; HMB, β-Hydroxy-β-Methylbutyrate; HMB-ONS, HMB-Enriched Oral Nutritional Supplements; HMO, Clalit Health Maintenance Organization; ICD-9, International Classification of Diseases, Ninth Revision; IGF-1, Insulin-like Growth Factor 1; ILS, Israeli New Shekels; IQR, Interquartile Range; MD, Mean Difference; MOH, Ministry of Health; MUST, Malnutrition Universal Screening Tool; NOURISH, Nutrition effect On Unplanned Readmissions and Survival in Hospitalized patients; ONS, Oral Nutritional Supplements; OR, Odds Ratio; PPN, Peripheral Parenteral Nutrition; PSM, Propensity Score Matching; ROI, Return on Investment; RR, Relative Risk; S-ONS, Standard Oral Nutritional Supplements; SES, Socioeconomic Status; SHIELD, Strengthening Health In ELDerly through nutrition; SMD, Standard Mean Difference; TPN, Total Parenteral Nutrition.
